# Large-area growth of multi-layer hexagonal boron nitride on polished cobalt foils by plasma-assisted molecular beam epitaxy

**DOI:** 10.1038/srep43100

**Published:** 2017-02-23

**Authors:** Zhongguang Xu, Hao Tian, Alireza Khanaki, Renjing Zheng, Mohammad Suja, Jianlin Liu

**Affiliations:** 1 Quantum Structures Laboratory, Department of Electrical and Computer Engineering, University of California, Riverside, California 92521, USA

## Abstract

Two-dimensional (2D) hexagonal boron nitride (h-BN), which has a similar honeycomb lattice structure to graphene, is promising as a dielectric material for a wide variety of potential applications based on 2D materials. Synthesis of high-quality, large-size and single-crystalline h-BN domains is of vital importance for fundamental research as well as practical applications. In this work, we report the growth of h-BN films on mechanically polished cobalt (Co) foils using plasma-assisted molecular beam epitaxy. Under appropriate growth conditions, the coverage of h-BN layers can be readily controlled by growth time. A large-area, multi-layer h-BN film with a thickness of 5~6 nm is confirmed by Raman spectroscopy, scanning electron microscopy, X-ray photoelectron spectroscopy and transmission electron microscopy. In addition, the size of h-BN single domains is 20~100 μm. Dielectric property of as-grown h-BN film is evaluated by characterization of Co(foil)/h-BN/Co(contact) capacitor devices. Breakdown electric field is in the range of 3.0~3.3 MV/cm, which indicates that the epitaxial h-BN film has good insulating characteristics. In addition, the effect of substrate morphology on h-BN growth is discussed regarding different domain density, lateral size, and thickness of the h-BN films grown on unpolished and polished Co foils.

Two-dimensional (2D) hexagonal boron nitride (h-BN) has received a great deal of attention due to its remarkable properties^1^,^2^,^3^,^4^, such as large band gap (~6.0 eV), high thermal conductivity, excellent thermal and chemical stability, and its significant potential as an indispensable dielectric layer of building blocks in high-performance 2D integrated electronics and photonics^5^,^6^,^7^,^8^,^9^,^10^. Among those efforts, 2D h-BN, which has a small lattice mismatch (1.7%) with graphene, and an atomically smooth surface that is relatively free of dangling bonds and trapped charges^5^, has been recently demonstrated to be an ideal encapsulation for graphene and other 2D materials^8^,^11^,^12^,^13^,^14^,^15^. In addition, h-BN has been used as a gate dielectric to greatly enhance the performance of graphene electronics^5^,^10^,^16^. To realize the technological potential of h-BN, it is essential to synthesize large-area, high-quality h-BN thin films through a scalable and controllable method. So far, tremendous efforts have been made to obtain large-area and high-quality h-BN films. Mechanical cleavage^17^ and liquid exfoliation^18^ can provide micrometer-sized, atomic-layer-thick h-BN flakes, but they are not suitable for large-area production due to the difficulty of controlling the thickness and size of the materials. Much progress has been made by chemical vapor deposition (CVD) of h-BN growth on various substrates, including Cu^4^,^19^,^20^,^21^, Ni^22^,^23^, Fe^11^,^24^, Ru^25^ and Pt^26^,^27^. In addition, other methods, such as physical vapor deposition (pulsed laser deposition^28^, reactive magnetron sputtering^25^, ion beam sputtering deposition^21^,^29^) and co-segregation^30^,^31^, have been attempted. Nevertheless, there is still much to be done in each one of these methods in order to controllably and scalably synthesize high-quality h-BN thin films.

Most recently, molecular beam epitaxy (MBE) has been used to synthesize h-BN films on cobalt (Co) thin film and nickel (Ni) foil substrates^32^,^33^,^34^. As an alternative approach to other methods, MBE can provide precise control over the growth conditions thanks to its ultra-high vacuum environment, atomic layer epitaxy accuracy and controllability, instant introduction and control of multiple sources, ease of doping of materials and *in-situ* layer-by-layer characterization. In addition, MBE is promising for the *in-situ* growth of vertically stacked heterostructures with defect-free interfaces^35^, which can bring about new opportunities to develop integrated 2D nanoelectronics, nanophotonics and spintronics^36^. On the other hand, although h-BN has been grown on Ni foils by MBE^33^,^34^, the growth mechanism remains elusive, moreover, no electrical and dielectric properties of those MBE grown h-BN films have been evaluated. In this paper, we report MBE growth of large-area, multi-layer h-BN films on polished Co foils for the first time. The growth mechanism is discussed based on the effect of substrate surface morphology on h-BN growth, and electrical and dielectric properties are characterized.

## Experiments

A re-designed Perkin-Elmer MBE system was used for sample growth. A Knudsen effusion cell filled with B_2_O_3_ powder (Alfa Aesar, 99.999%) was used as boron (B) source. Nitrogen plasma (Airgas, 99.9999%) generated by an electron cyclotron resonance (ECR) system and high-purity ammonia (American Gas Group, 99.9995%) were used as nitrogen (N) sources (Supporting Information).

Co foils (0.1 mm thick, 99.995%) from Alfa Aesar were cut into 1 cm × 1 cm pieces as substrates. These pieces were degreased with acetone and IPA and rinsed in deionized (DI) water prior to use. Besides these unpolished substrates, polished substrates were mainly used in this experiment. The Co foil surface polishing was performed on an SBT 920 Lapping and Polishing workstation (details in Supporting Material). These Co foil substrates were cleaned with diluted hydrochloric acid (10%) to remove residual native oxide layer, rinsed with DI water, blown dry, and immediately loaded into the MBE chamber. The substrates were heated to 850 °C and annealed at this temperature under a 10-sccm (standard cubic centimeters per minute) flow of hydrogen gas for 10 minutes. Then, h-BN growth was started at the same substrate temperature. During the growth, B cell temperature was maintained at 1150 °C; N_2_ gas flowed at 5 sccm through an ECR source, and NH_3_ gas at a flow rate of 5 sccm was also introduced to the chamber through a needle valve. The ECR current was set at 60 mA with a power of 228 W, and the growth took 900 s for a reference sample on an unpolished Co foil (Sample A), and 450 s, 900 s and 1800 s on polished Co foils for Samples B, C, and D, respectively. Finally, the samples were cooled to room temperature at a rate of 10 °C/min. Table S1 (Supporting Materials) gives a summary of the growth conditions.

Raman characterizations were performed using a HORIBA LabRam system equipped with a 50-mW, 532-nm green laser. Scanning electron microscopy (SEM) images were acquired using an XL30-FEG SEM system. X-ray photoelectron spectroscopy (XPS) characterization was conducted using a Kratos AXIS ULTRA XPS system equipped with an Al Kα monochromatic X-ray source and a 165-mm mean radius electron energy hemispherical analyzer. Atomic force microscopy (AFM) images were obtained using a Veeco D5000 AFM system. Transmission electron microscopy (TEM) images and selected area electron diffraction (SAED) patterns were acquired using a FEI Titan Themis 300 STEM. Plan-view TEM sample was prepared using a PMMA-assisted transfer method. After spin-coating with PMMA, the sample was submerged in FeCl_3_ solution to etch away the Co metal layer. The film was then transferred onto a carbon-coated Cu TEM grid and treated with acetone and DI water to remove the PMMA. Samples transferred onto SiO_2_-coated Si substrates were obtained using the same PMMA-assisted method.

Co(foil)/h-BN/Co(contact) capacitor devices were fabricated by a standard photolithography and lift-off process. A Co layer of 100 nm was patterned as top square contacts with an edge length of 20 μm, 50 μm and 100 μm on the surface of as-grown h-BN film. Reactive ion etching (RIE) was performed with a 50-sccm SF_6_ plasma, under a power of 50 W, and for 15 seconds to etch the h-BN film between devices, which ensured isolation of different devices on the same substrate. The current-voltage (I-V) characteristics were obtained by an Agilent 4155 C semiconductor parameter analyzer equipped with probing tips having a diameter of 5 μm (Signatone, SE-TL).

## Results and Discussion

**Figure 1:**
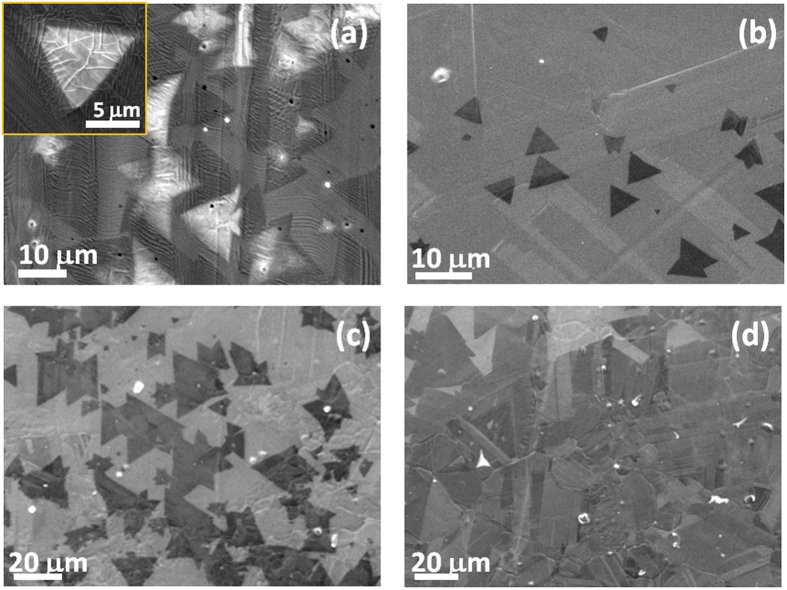
SEM images of h-BN films grown on (**a**) unpolished foil for 900 s (Sample A), and on polished foils for (**b**) 450 s (Sample B), (**c**) 900 s (Sample C) and (**d**) 1800 s (Sample D). The inset in (**a**) is a magnified image of an h-BN flake on Sample A.

Both unpolished and polished Co foils were used as substrates. The substrate cleaning, treatment and polishing procedures are described in the Experimental section in detail. The surface morphology comparison between unpolished and polished Co foils is shown in Figure S1, and discussed in the Supporting Material. Within a scanned area of 50 × 50 μm^2^, the root mean square (RMS) roughness of the polished Co foil is about 11 nm, compared to 231 nm for the unpolished Co foil. Figure 1 shows SEM images of the four as-grown samples (additional SEM images in Figure S2). All samples show h-BN features that are darker than their substrates in the images. White triangular h-BN domains are also observed on Sample A, which was grown on unpolished Co (Fig. 1a). The inset of Fig. 1a shows a magnified SEM image of a typical white triangle, which should be multilayer h-BN stacks^21^,^24^. This result suggests that the MBE growth of h-BN is not self-limited^21^. Wrinkles are also evident on the surface, which represent the conformal growth of the film across very rough Co surface with ridges and valleys. In other words, these winkles are not induced by thermal strain effect as often observed from those films produced by fast substrate cooling^37^ because we used very low cooling rate, the stress originated by different thermal expansion coefficients between the film and substrate can be readily relaxed without causing the formation of wrinkles. This is further proved for the films on polished samples where no wrinkles are observed. For the samples grown on polished Co, as the growth time increases from 450 s to 1800 s, the morphology of h-BN film evolves from discrete flakes into continuous film, as shown in the SEM images in Fig. 1b–d. At a growth time of 450 s, Sample B exhibits sparse h-BN flakes in random domain orientations with various edge sizes from a few micrometers to a few tens of micrometers (up to 40 μm), which indicates that the nucleation sites are constantly formed and the initially formed smaller seeds grow into larger domains as the growth time elapses. Longer growth time of 900 s led to denser and relatively larger h-BN flakes, as well as conjoined h-BN domains (Figure S2c) on Sample C. However, flakes with smaller sizes can still be seen at this stage, further proving that new seeds have been formed continuously on the exposed Co surface. After the growth is extended to 1800 s, continuous h-BN film is finally formed on most of the Co surface except the edge areas of the sample, where discrete flakes still exist. The single domain boundaries can be easily identified (Figure S2d) and the single domain size can be estimated to be in the range of 20~100 um. H-BN flakes growing across Co grain boundaries are also frequently observed, which suggests that h-BN growth is surface-mediated under these conditions^19^,^21^.

In order to assess the surface morphology effect on the nucleation and growth of h-BN, we compare SEM and AFM images of Sample A (Fig. 1a and Figure S3) and Sample C (Fig. 1c and Figure S4), showing h-BN films on unpolished and polished Co foils, respectively, grown under the same growth conditions. In general, the h-BN grown on polished Co foil has larger single-domain size and more uniform thickness, compared with the h-BN grown on unpolished Co foil. It has been proposed that one key approach to grow large, single-crystalline h-BN domains is to reduce the nucleation density at the early stage of growth^20^. The defects, such as grain boundaries, rough edges, uneven grooves, and impurities on unpolished Co substrates provide relatively lower activation energy for h-BN to nucleate compared with smooth regions^36^. Since polishing process can remove a large amount of these defects, the nucleation sites are significantly reduced and more evenly distributed on polished Co surfaces. Thus, the h-BN domains grown on polished Co are more uniform in both domain size and thickness, compared with their counterparts on unpolished Co. The low nucleation density decreases the competition for impinging atoms among different seeds locally, which helps maintain the same growth conditions for different seeds. Farther apart seeds grow at the same rate and are evenly spread. After the initial h-BN nucleation, smooth polished Co surface provides lower kinetic energy barrier and longer diffusion length for B and N atoms to diffuse. These atoms with high kinetic energy can diffuse across a large area, and react and settle at the active edges of BN seeds, which continue to grow into large, single-crystalline domains. In contrast, on unpolished Co surfaces with a larger kinetic energy barrier and lower diffusion length caused by rough surface, B and N atoms tend to accumulate locally and form adlayers, rather than diffuse across the barrier. The formation of adlayers can be also associated with defects from rough substrate, which would cause strain in the existing layers, leading to further nucleation on top of the existing layers. Our result proves that it is indeed important to decrease the number of nucleation sites to achieve large domain sizes. In other words, there is a great possibility to achieve high-quality h-BN films with large-size and single-crystalline domains on smooth Co foil substrates.

**Figure 2:**
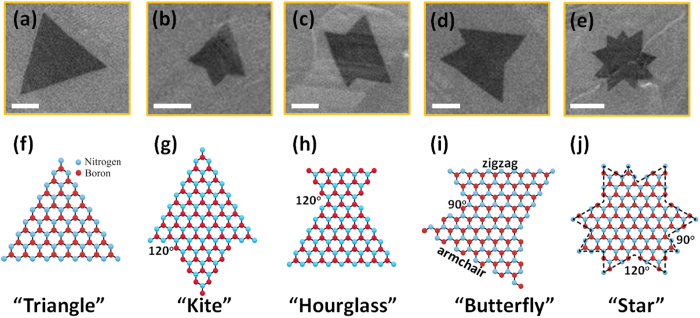
SEM images paired with schematic illustrations of h-BN domains with different atomic arrangements: (**a**,**f**) triangle shape, (**b**,**g**) “kite” shape, (**c**,**h**) “hourglass” shape, (**d**,**i**) “butterfly” shape and (**e**,**j**) multi-apex-star shape. Scale bars in (**a**–**e**) are 5 μm.

It is interesting to note that besides regular triangular domains, other complex polygons with well-defined edges are observed as the initial form of h-BN flakes among all samples on polished Co substrates. Figure 2a–e show SEM images of these flakes, and Fig. 2f–j show one-to-one corresponding possible schematic illustrations of their atomic arrangements: triangle shape, “kite” shape, “hourglass” shape, “butterfly” shape and multi-apex-star shape, respectively. From theoretical calculations^38^, the triangular h-BN domain is single crystal with nitrogen-terminated zigzag edges due to energetic preferences, as shown in Fig. 2a,f. Figure 2b,g show a kite-shape domain composed of two triangles with alternating nitrogen-terminated edges and boron-terminated edges^19^. Hourglass-shape domain is composed of two similar quadrangles with nitrogen-terminated edges and boron-terminated edges, as shown in Fig. 2c,h. In addition, mutually perpendicular edges are observed from butterfly-shape domain (Fig. 2d,i) and multi-apex-star-shape domain (Fig. 2e,j), which indicates the connection of one zigzag edge with a neighboring armchair edge from simple geometry. Although the growth mechanism of complex domain structures remains elusive, the two merging modes, namely, point-to-edge and edge-to-edge modes^39^ can be a possible explanation. For example, as the nucleation sites are close enough to one another, the coalescence between the domains would occur when they grow larger, resulting in multifaceted shapes.

**Figure 3:**
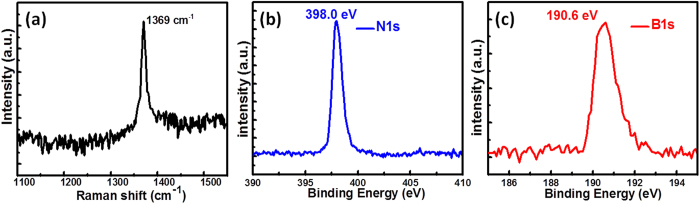
(**a**) Raman spectrum of as-grown h-BN film (Sample D). XPS spectra of (**b**) N1s and (**c**) B1s peaks.

Based on the continuous film of Sample D, further characterizations have been performed to assess the h-BN. Figure 3a shows Raman spectrum of the as-grown h-BN film. A single peak at 1368 cm^−1^ is assigned to the E_2g_ vibration mode of h-BN^20^. In addition, the full width at half maximum (FWHM) of this Raman peak is only 11 cm^−1^, which is indicative of high-crystalline h-BN film^33^. XPS spectra in Fig. 3b and c show an evident peak for B 1 s and N 1 s at 190.6 and 398.0 eV, respectively, further confirming the existence of the B-N bond^4^,^11^. The B/N elemental ratio can be extracted as ∼1:1.06 based on the integral intensities of the characteristic peaks, which suggests an almost equal composition of B and N elements.

**Figure 4:**
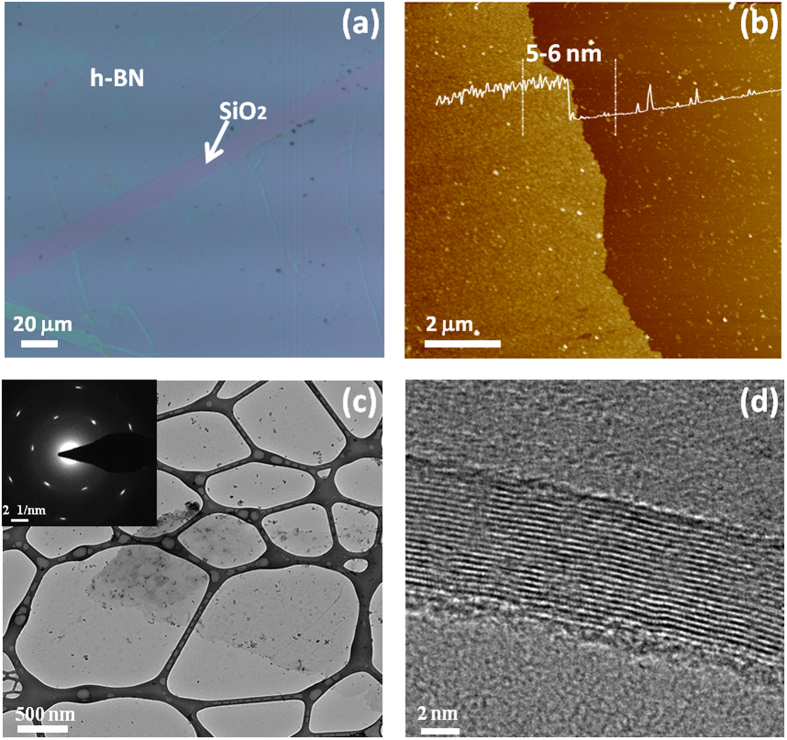
(**a**) Optical micrograph and (**b**) AFM image of the transferred h-BN film (Sample D) on SiO_2_. (**c**) Plan-view TEM image of the transferred h-BN film on a carbon-coated Cu TEM grid. Inset is an electron diffraction pattern, showing six-fold symmetry of the h-BN film. (**d**) High-resolution TEM image showing multi-layer structure of the h-BN film near its edge.

Figure 4a shows an optical microscopy image of the h-BN film transferred onto a SiO_2_/Si substrate, showing a large-size continuous film with a clear contrast from the SiO_2_/Si substrate. The surface morphology of the h-BN film was further characterized by AFM, which is shown in Fig. 4b. The height profile of a scanned line indicates that the h-BN has a thickness of 5~6 nm on average for 1800-s growth. It is worth noting that the thickness of the h-BN flakes in Sample C (900-s growth) is estimated to be 2.2 nm (Figure S4), which suggests that h-BN growth may start with nucleation sites of a few layers and then grow into large domains^40^. Considering the thickness and coverage evolution from Sample C to Sample D, it indicates that initial flakes grow laterally as well as vertically into multilayer film as the growth time lapses. Figure 4c shows a plan-view TEM image of the transferred h-BN film. A continuous thin film is observed. The SAED pattern of the h-BN thin film is seen in the inset of Fig. 4c, showing a clear hexagonal set of diffraction spots. The in-plane lattice constant can be estimated as approximately 2.47~2.5 Å, which is in good agreement with that of h-BN^22^. High resolution TEM image (Fig. 4d) shows the lattice fringes of the h-BN film with an interlayer distance of 0.33 nm, which matches previously reported values of h-BN^11^,^31^. The thickness is measured to be ~6 nm, which is consistent with the AFM results.

**Figure 5:**
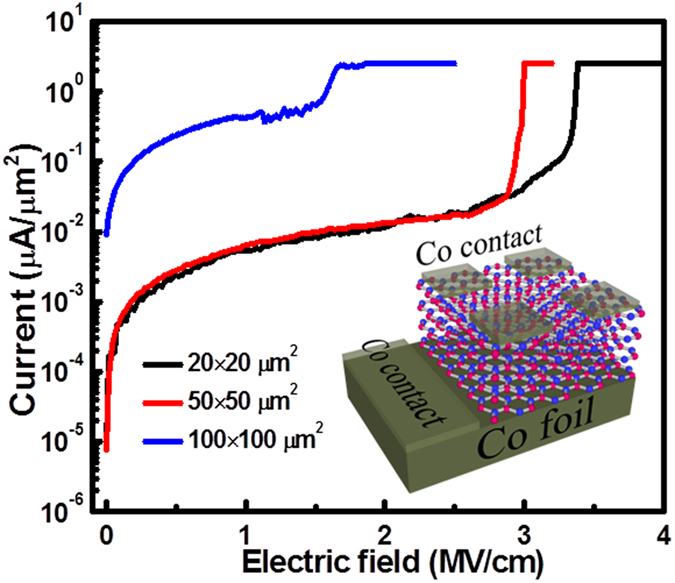
Current density-electric field characteristics of Co(foil)/h-BN/Co(contact) devices with different contact sizes. Inset shows a schematic of the devices.

To evaluate the insulating property of the h-BN film, electrical breakdown characterization was performed on Co(foil)/h-BN/Co(metal contact) capacitor devices based on Sample D. Figure 5 shows typical curves of current density versus electric field from square-shaped devices with three different edge lengths of 100, 50, and 20 μm, respectively. The 100-μm device shows significant leakage without any evident breakdown point, which can be originated from pinholes, grain boundaries or significant defects (Figure S2d), leading to a conducting behavior. In contrast, devices with 50-mm and 20-μm edge lengths show much-improved insulating characteristics although there were still many of the tested devices showing similar behavior to that of the 100-μm-sized devices due to the same above-mentioned factors. In addition, it is worth to note that these smaller devices have similar leakage current at lower bias compared with those based on mechanically exfoliated BN films^41^. The hard breakdown phenomenon can be observed in the range of 3.0~3.3 MV/cm, which is comparable with the values from the devices based on h-BN films grown by CVD^8^,^11^,^42^ and exfoliated h-BN films^43^. In addition to the vertical electrical characterizations, in-plane electrical characterizations were also carried out on a transferred h-BN thin film of Sample D on SiO_2_ (Figure S5). Currents are negligible under a voltage sweep across pairs of separated metal contacts placed on top of the film. These results suggest good insulating characteristics of the h-BN film grown by MBE.

## Conclusion

We demonstrated large-area growth of multi-layer h-BN films on mechanically polished Co foils using plasma-assisted MBE. Time-dependent h-BN growth indicates that the coverage of h-BN domains with various polygon shapes on the surface increases as the increase of the growth time. The dielectric properties of as-grown h-BN film with a thickness of 5~6 nm are evaluated by characterization of Co(foil)/h-BN/Co(contact) capacitor devices. Breakdown electric field is estimated to be 3.0~3.3 MV/cm, which indicates that the epitaxial h-BN film has good insulating characteristics. In addition, by comparing the results of h-BN growth on unpolished and polished Co foils, it is concluded that the morphology of Co foil surface affects h-BN growth with respect to domain density, lateral size and thickness since the control of the density of nucleation sites plays a key role on the growth of h-BN films.

## Additional Information

**How to cite this article**: Xu, Z. *et al*. Large-area growth of multi-layer hexagonal boron nitride on polished cobalt foils by plasma-assisted molecular beam epitaxy. *Sci. Rep.* **7**, 43100; doi: 10.1038/srep43100 (2017).

**Publisher's note:** Springer Nature remains neutral with regard to jurisdictional claims in published maps and institutional affiliations.

## Supplementary Material

Supplementary Information
