# Supplementary material for: Large-area growth of multi-layer hexagonal boron nitride on polished cobalt foils by plasma-assisted molecular beam epitaxy
Source: Sci Rep. 2017 Feb 23;7:43100. doi: 10.1038/srep43100 (PMC5322373; doi:10.1038/srep43100)
Supplement: Supplementary Information [file srep43100-s1.pdf]

Supplementary information

Large-area growth of multi-layer hexagonal boron nitride on polished cobalt foils by plasma-assisted molecular beam epitaxy

Zhongguang Xu, Hao Tian, Alireza Khanaki, Renjing Zheng, Mohammad Suja, Jianlin Liu *

Z. Xu, H. Tian, A. Khanaki, R. Zheng, M. Suja, Prof. J. L. Liu

Quantum Structures Laboratory,

Department of Electrical and Computer Engineering,

University of California,

Riverside, California 92521, USA

*Corresponding E-mail: jianlin@ece.ucr.edu

Table S1. Summary of sample growth condition

Step	Parameters	Sample A	Sample B	Sample C	Sample D
Pre-growth Annealing	Temperature	850 ^oC	850 ^oC	850 ^oC	850 ^oC
	Hydrogen gas flow	10 sccm	10 sccm	10 sccm	10 sccm
	Duration	10 minutes	10 minutes	10 minutes	10 minutes
H-BN Growth	Substrate	*Unpolished Co foil*	*Polished Co foil*	*Polished Co foil*	*Polished Co foil*
	Substrate temperature	850 ^oC	850 ^oC	850 ^oC	850 ^oC
	Boron cell temperature	1150 ^oC	1150 ^oC	1150 ^oC	1150 ^oC
	Ammonia gas flow	5 sccm	5 sccm	5 sccm	5 sccm
	Nitrogen gas flow	5 sccm	5 sccm	5 sccm	5 sccm
	Nitrogen ECR current	60 mA	60 mA	60 mA	60 mA
	Growth duration	900 seconds	450 seconds	900 seconds	1800 seconds

Figure S1. Photographs of (a) unpolished Co foil and (b) polished Co foil. Scratched lines are visible on unpolished Co foil, while polished Co foil exhibits a shining surface. Optical microscopy images of (c) unpolished Co foil and (d) polished and high-temperature annealed Co foil. AFM images of (e) unpolished and (f) polished and high-temperature annealed Co foils. The rough and deep parallel grooves on the surface of unpolished Co foil can be clearly seen from the optical microscopy and AFM images in (c) and (e), respectively. After mechanical polishing and high-temperature annealing, significant improvement of the surface flatness is evident, as revealed by the optical microscopy and AFM images in (d) and (f), respectively. Within a scanned area of $50 \times 50 \mu m^2$, the root mean square (RMS) roughness of the polished Co foil is 11 nm, compared to 231 nm for the unpolished Co foil. The inset in (f) shows an AFM image of a zoomed-in area with a RMS roughness of ~3 nm.

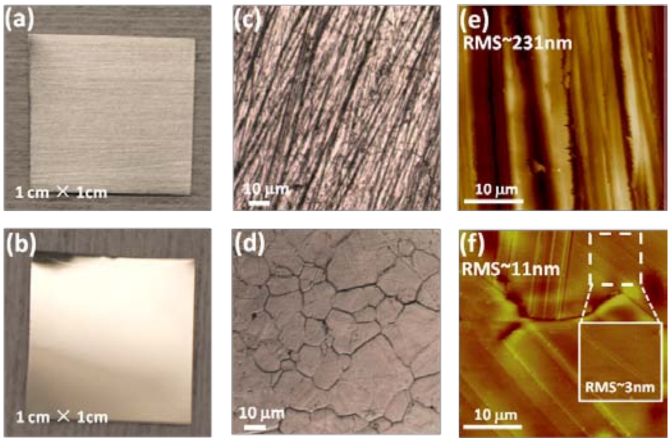

Figure S2. Additional SEM images of (a) Sample A, (b) Sample B, (c) Sample C, (d) Sample D. These images were taken with lower magnification compared to those in the main text in order to show h-BN coverage in larger areas. Insets in (c) and (d) show their corresponding magnified SEM images.

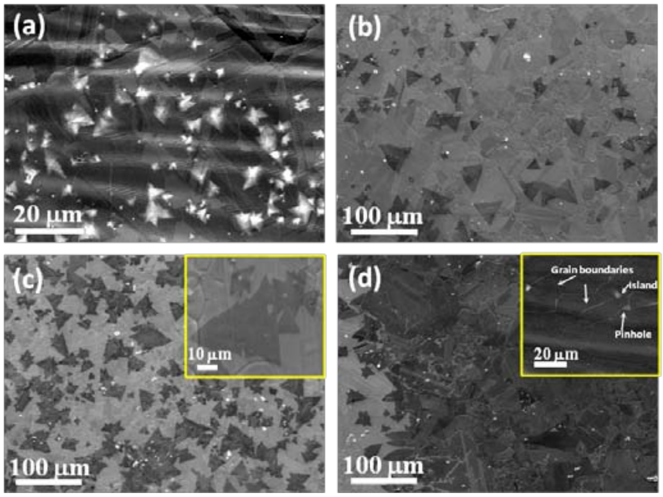

Figure S3. a) SEM and b) AFM images of an individual h-BN domain from Sample A after transferred on an SiO_2/Si substrate. Inset of (b) shows the height profile.

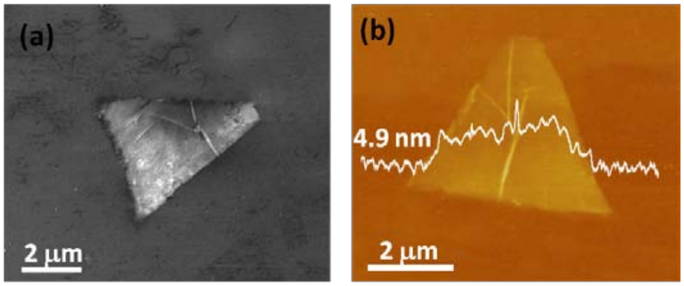

Figure S4. a) SEM, b) AFM and c) Raman mapping of an individual h-BN domain from Sample C after transferred on an SiO_2/Si substrate. Inset of (b) shows the height profile. Inset of (c) is a typical Raman spectrum of transferred h-BN film.

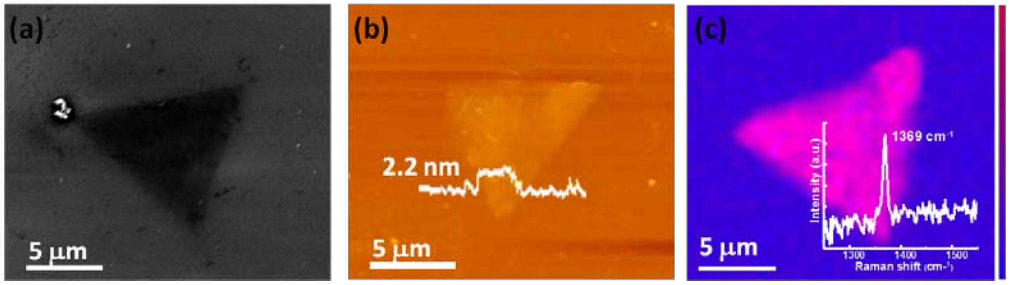

Figure S5. Current-voltage characteristic of a transferred h-BN film on SiO_2/Si substrate. The inset shows an SEM image of the device. A 100-nm Co layer was deposited and standard photolithography and lift-off process were used to define the top contacts. There were negligible current flowing as the voltage was scanned across the top/bottom metal contact and right/left metal contact pairs with a scanned range of voltage (-5/+5), indicating the insulating nature of h-BN.

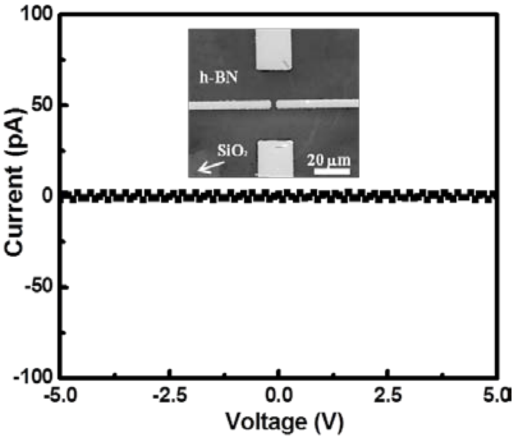

Preparation of polished Co foils

The Co foil surface polishing was performed on an SBT 920 Lapping and Polishing workstation (Fig. S6). Co foil pieces of 1 cm × 1 cm were mounted on a model 155 lapping and polishing fixture and placed against the workstation. The polishing process was divided into two stages: lapping and polishing. During lapping process, 8-inch abrasive discs of 30, 15, 6 and 3 µm were utilized to remove subsurface damage (scratches and grooves) caused by sawing or grinding, producing a smooth, flat, unpolished surface (generally less than 2.5-µm uniformity). The

lapping plate usually rotates at a low speed (<80 rpm). Then, 2-µm silicon oxide powder, 1-µm and 0.3-µm alfa aluminum powder, and 0.06-µm colloidal silica suspension were used sequentially to fine polish Co and produce a scratch-free, specular surface. Polishing is typically done at very low speeds (<50 rpm) using polishing clothes. After polishing, the polished foils were sonicated by putting in acetone, IPA and DI water, respectively, to remove possible residues. SEM and EDX measurements have been carried out to examine the morphology and elemental survey of polished foils, showing clean and flat surface together with the EDX peak of Co only (Fig. S7).

Figure S6. Schematic of the polishing machine.

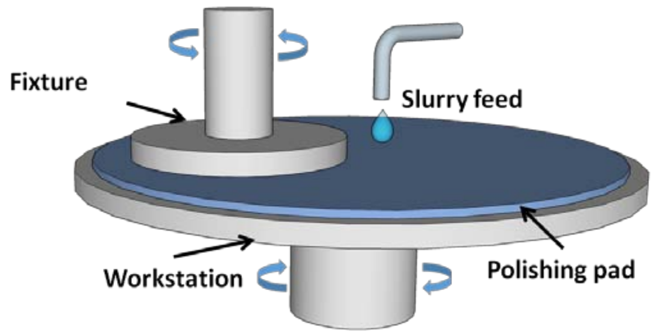

Figure S7. a) SEM image of the polished Co foil and b) its corresponding EDX spectrum.

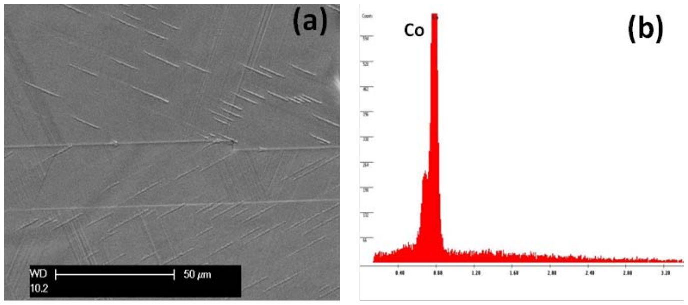

Discussion on nitrogen source: N_2 molecules have a strong N-N bond, which is very difficult to break up. Although electron-cyclotron resonance (ECR) or radio frequency (RF) plasma can be used to excite nitrogen molecules into active N atoms or free-radicals, the efficiency of the related growth is quite low in general [1-2]. In addition, some reports suggest that accelerated ionized nitrogen species would result in structural defects in epitaxial films [3]. On the other hand, it is relatively easy to dissociate NH_3 and especially, the molecular break-up process can be easily achieved on a heated substrate surface. In addition, hydrogen from decomposition of NH_3 can effectively help remove the oxygen from growth environment. However, using high flux of NH_3 may cause safety issue and high H load for the MBE chamber [4]. With these

considerations, the mixture of nitrogen and ammonia as nitrogen sources was selected, which led to controllable growth.

Transferring process: PMMA (495 A4) was used as a spin-coating material on the sample. Spin-coating is for 45s at 4000 rmp, after the sample was baked at 170 ^{o}C for 10 minutes. Float the sample on the etching solution (mixing 3% H_2O_2 and 37%HcL at a ratio of 3:1) for more than 12-hour to remove the Co foil. Take the film out of the solution using SiO_2 substrate and dry it at room temperature or 60 degree C for 12 hours to make sure all the water comes out. Put it into Acetone or PG remover solvent to remove PMMA. After that, anneal the sample at 400 ^{o}C for at least 3 hours in O_2 ambient.

References

[1] Paisley M. J., Sitar Z., Van B. & Davis R. F. J. Growth of boron nitride films by gas molecular-beam epitaxy. Vac. Sci. Technol. B 8, 323(1990).

[2] Tsai C. L., Kobayashi Y., Akasaka T. & Kasu M. Molecular beam epitaxial growth of hexagonal boron nitride on Ni(1 1 1) substrate. J. Cryst. Growth 311, 3054-3057(2009).

[3] Grant V. A., Campion R. P., Foxon C. T., Lu W., Chao S. & Larkins E. C. Optimization of RF plasma sources for the MBE growth of nitride and dilute nitride semiconductor material. Semicond. Sci. Technol. 22, 15-19(2007).

[4] Skierbiszewski C., Wasilewski Z. R., Grzegory I. & Porowski S. N. Nitride-based laser diodes by plasma-assisted MBE—From violet to green emission. J. Cryst. Growth 311, 1632-1639(2009).
